# Immune Checkpoint Blockade – How Does It Work in Brain Metastases?

**DOI:** 10.3389/fnmol.2019.00282

**Published:** 2019-11-21

**Authors:** Mihaela Lorger, Tereza Andreou, Christopher Fife, Fiona James

**Affiliations:** ^1^Institute of Medical Research at St. James’s, School of Medicine, University of Leeds, Leeds, United Kingdom; ^2^Leeds Teaching Hospitals NHS Trust, Leeds, United Kingdom

**Keywords:** brain metastases, immunotherapy, immune response, immune checkpoint, extracranial tumor

## Abstract

Immune checkpoints restrain the immune system following its activation and their inhibition unleashes anti-tumor immune responses. Immune checkpoint inhibitors revolutionized the treatment of several cancer types, including melanoma, and immune checkpoint blockade with anti-PD-1 and anti-CTLA-4 antibodies is becoming a frontline therapy in metastatic melanoma. Notably, up to 60% of metastatic melanoma patients develop metastases in the brain. Brain metastases (BrM) are also very common in patients with lung and breast cancer, and occur in ∼20–40% of patients across different cancer types. Metastases in the brain are associated with poor prognosis due to the lack of efficient therapies. In the past, patients with BrM used to be excluded from immune-based clinical trials due to the assumption that such therapies may not work in the context of “immune-specialized” environment in the brain, or may cause harm. However, recent trials in patients with BrM demonstrated safety and intracranial activity of anti-PD-1 and anti-CTLA-4 therapy. We here discuss how immune checkpoint therapy works in BrM, with focus on T cells and the cross-talk between BrM, the immune system, and tumors growing outside the brain. We discuss major open questions in our understanding of what is required for an effective immune checkpoint inhibitor therapy in BrM.

## Introduction

Brain metastases (BrM) are the most frequent intracranial tumors, representing an unmet clinical need with poor prognosis. They develop in 20–40% of metastatic cancer patients and mostly originate from lung cancer, breast cancer and melanoma (Gerrard and Franks, 2004; DeAngelis, 2008; Valiente et al., 2018; Doron et al., 2019). Until recently, treatment options have been restricted to radiotherapy and surgery, and the median overall survival (OS) after combination of these therapies is below 1 year (Puzanov et al., 2013; Ajithkumar et al., 2015). Patients with BrM are frequently excluded from clinical trials (Ajithkumar et al., 2015). Consequently, BrM are understudied at the clinical and preclinical level, and the treatment options for BrM are commonly lagging behind.

Programmed Death 1 (PD-1) and Cytotoxic T-lymphocyte Associated Protein 4 (CTLA-4) are immune-inhibitory receptors (immune checkpoints) expressed mainly on T cells, and their inhibition with function-blocking antibodies has been shown to enhance anti-tumor T cell responses (Walker and Sansom, 2011; Kamphorst and Ahmed, 2013). Antibodies targeting CTLA-4 (Ipilimumab) and PD-1 (Nivolumab, Pembrolizumab) have shown a great promise for the treatment of different cancers. Moreover, there is now substantial evidence for the efficacy of both anti-CTLA-4 and anti-PD-1 therapy in BrM. A handful of retrospective and prospective clinical studies indicated activity of ipilimumab in melanoma BrM with 16–25% intracranial response rate, but also suggested that only a subgroup of patients is likely to benefit (Margolin et al., 2012; Puzanov et al., 2013; Ajithkumar et al., 2015). Pembrolizumab and nivolumab showed ∼21% response rate in BrM in melanoma patients (Goldberg et al., 2016; Gonzalez-Cao et al., 2017; Long et al., 2017; Parakh et al., 2017). Intracranial activity was also observed in patients with non-small-cell lung cancer (NSCLC), reporting 33% objective response rate (Goldberg et al., 2016) and 47% disease control (Bidoli et al., 2016), respectively, as well as in renal cell carcinoma with a response rate of 18.7% in the central nervous system (CNS; De Giorgi et al., 2019). Two very recent clinical trials in drug-treatment naïve patients with melanoma BrM [ABC trial (Long et al., 2017) and CheckMate 204 trial (Tawbi et al., 2017)] reported a 46 and 52% intracranial response rate respectively following combined anti-PD-1 plus anti-CTLA-4 therapy. The ABC trial also demonstrated superior intracranial activity of combined PD-1 plus CTLA-4 blockade as compared to the PD-1 blockade alone. In summary this demonstrates clinical efficacy of immune checkpoint inhibitors in the brain in the context of metastatic disease.

It is now well accepted that the brain is an immune-specialized rather than immune-privileged environment. Importantly, the CNS contains several immunologically distinct compartments; while grafts implanted into the brain parenchyma or cortex display prolonged survival, those implanted into ventricles are readily rejected (Murphy and Sturm, 1923; Medawar, 1948; Thomas et al., 2008). The aim of this review is to discuss how immune responses in the context of immune checkpoint blockade and some other immunotherapies occur in BrM. We will focus on tumors located within the brain parenchyma – a brain compartment that seems to be the most restrictive/specialized in terms of immune reactions, and on T cells as critical mediators of antigen-specific immune responses. We will also discuss how the presence of extracerebral cancer lesions affects tumors located within the brain.

## Immune Crosstalk Between Brain Tumors and the Periphery

It has been observed almost 100 years ago that mouse sarcoma tumors, which would typically be rejected when transplanted under the skin in rats, grew efficiently within rat brain parenchyma (Shirai, 1921). The same was true for skin grafts transplanted into the brain; however, skin grafts in the brain were rejected if animals spontaneously rejected a graft of the same tissue growing in the skin (Medawar, 1948). Interestingly, several preclinical glioma models that grow aggressively in the brain, are spontaneously rejected when growing under the skin (Kida et al., 1983; Paul et al., 2000; Su et al., 2000; Barth and Kaur, 2009; Volovitz et al., 2011). Such spontaneous rejection of subcutaneous (s.c.) tumors is sufficient to induce rejection of intracranial tumors (Volovitz et al., 2011). Volovitz et al. (2011) termed the phenomenon where tumor that in one location is immune-resistant and in another location generates protective immunity “split immunity.” Importantly, the spread of immunological information in this context is unidirectional: while the immunity generated by s.c. tumors spreads to the intracranial tumors, intracranial tumors are incapable of spreading immunity to s.c. tumors (Volovitz et al., 2011).

The situation is somewhat different in the context of BrM which originate from extracranial tumors that are immunologically compatible with their host; simultaneous or prior subcutaneous growth of immunologically compatible melanoma tumors *per se* namely doesn’t impact intracranial growth of the same tumor (Lu et al., 2003; Taggart et al., 2018). Interestingly, however, the presence of extracranial tumor does have an effect on BrM in the context of immune-based therapies. Our lab recently demonstrated that immune checkpoint blockade with combined anti-PD-1 plus anti-CTLA-4 therapy inhibits B16 and Ret melanoma growth in the brain only if the mice are simultaneously bearing tumors of the same type under the skin, while the therapy failed in mice with intracranial tumors only (Taggart et al., 2018). The presence of extracranial tumor significantly increased the numbers of circulating effector CD8+ T cells in treated mice, implying that mounting of systemic anti-tumor immune responses underlies intracranial therapeutic efficacy. The PD-1 immune checkpoint plays a role primarily within the tumor microenvironment, where it inhibits T cell responses by binding to one of its ligands (Wei et al., 2018). As blood vessels are less permeable in intracranial than extracranial tumors (Lockman et al., 2010; Matthias et al., 2016), it is possible that anti-PD-1 blocking antibodies cannot reach intracranial tumors sufficiently to release T cells from PD-1 blockade, and therefore, efficient anti-tumor immune responses in the brain may rely on the release of tumor antigen-specific T cells from PD-1 inhibition within the extracranial tumor. The CTLA-4 immune checkpoint is upregulated on T cells following T cell receptor (TCR) engagement of antigen-bound major histocompatibility complex (MHC) on antigen presenting cells (APCs) during T cell priming in secondary lymphoid organs. CTLA-4 dampens TCR signaling through competition with the costimulatory molecule CD28 for binding to CD80 and CD86 on APCs (Wei et al., 2018). As discussed below, tumor antigens originating from the intracranial tumor may reach tumor-draining LNs insufficiently to induce substantial T cell priming, and therefore, efficient generation of anti-tumor immune responses against tumors in the brain may rely on T cell priming and the release of tumor antigen-specific T cells from CTLA-4 blockade within the extracranial tumor-draining LNs.

In line with our study focusing on immune checkpoint blockade (Taggart et al., 2018), another study in melanoma has shown inhibition of brain colonization by melanoma cell line once subcutaneous tumors of the same type have been rejected following intra-tumoral administration of IFNβ-expressing insect cells, but not when a different s.c. tumor type was rejected (Lu et al., 2003). This study also reported that a failure to reject s.c. tumors following treatment occurred in a small percentage of mice, and this correlated with efficient establishment of BrM. Another example of a cross-talk between extracranial and intracranial tumors in the context of immunotherapy was reported in a breast cancer model; a rejection of orthotopic EMT6 breast carcinoma tumors through peri-tumoral administration of CpG oligodeoxynucleotides (ODN) prevented intracranial growth of the same cell line (Xiong et al., 2008). Taken together, these data suggest that while there is a barrier to the immune-based rejection of tumors growing in the brain as the only tumor site, a prior development of effective immunity against extracranial tumor sharing the same tumor antigens unleashes effective immune attack on BrM. In line with that, a strong concordance between systemic and intracranial responses to pembrolizumab has been reported in melanoma and NSCLC patients with BrM following the initial treatment period (Goldberg et al., 2016).

## What Restricts Immune Responses Against Tumors in the Brain?

**FIGURE 1:**
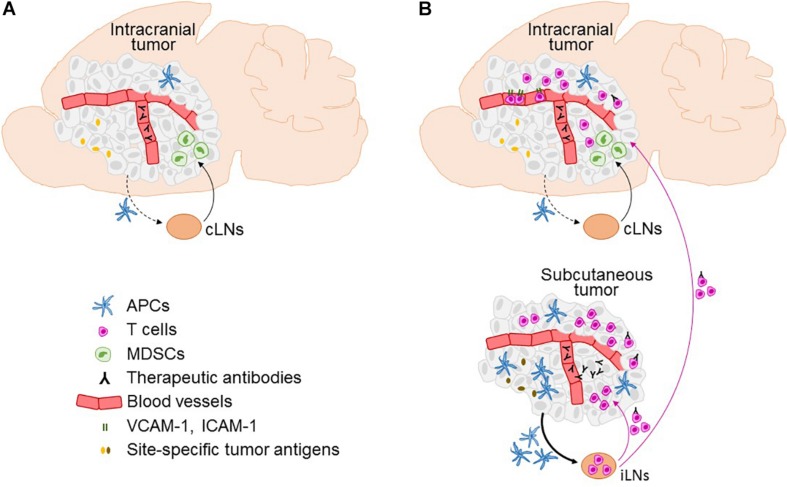
Factors affecting the efficacy of immunotherapy in the brain and the role of the extracranial disease. Immune microenvironment in intracranial tumors in the context of immunotherapies is depicted in the absence **(A)** and presence **(B)** of extracranial tumor, including infiltration of T cells, expression of T cell entry receptors ICAM-1 and VCAM-1 on blood vessels, and the access of therapeutic antibodies. Furthermore, the figure illustrates factors that differ between intracranial and extracranial tumor, as well as their respective draining lymph nodes (LNs), and are potentially involved in limiting the ability of intracranial tumor to mount effective systemic anti-tumor immune responses. This includes differences in the numbers of antigen presenting cells (APCs), lower efficiency of migration of APCs from the intracranial tumor to the cervical LNs (cLNs; dotted black line) as compared to the APC migration from the extracranial (subcutaneous) tumor to the inguinal LNs (iLNs; full line), an increased presence of myeloid derived suppressor cells (MDCSs) in intracranial as compared to the extracranial tumor, potential differences in tumor antigen expression at intracranial versus extracranial site, and lack of penetration of therapeutic antibodies into intracranial tumor in the absence of extracranial tumor. A potential transport of therapeutic antibodies on extracranial tumor-activated T cells into the brain following immune checkpoint inhibitor therapy is also depicted.

Cervical LNs (cLNs) are intracranial tumor-draining LNs, as this is where the antigens originating from intracranial tumors are predominantly found and where T cell proliferation is induced following intracranial tumor growth (Calzascia et al., 2005; Thomas et al., 2008). Characteristics of tumor-draining LNs may critically influence immune responses. Thomas et al. (2008) compared tumor antigen drainage following implantation of the same number of cancer cells into the ventricles, under the skin, and into the brain parenchyma in a small (0.3 uL) or a large volume (5 uL) – the latter being observed to result in an overflow of cancer cells into the ventricles. Intraparenchymal cancer cell injection in a small volume resulted in a significantly stronger accumulation of tumor antigens in parotid and deep cLNs as compared to the other modes of cancer cell injection. The stronger drainage of antigens to the cLNs correlated with a significantly higher number of myeloid derived suppressor cells and decreased number of CD8+ T cells in brain tumors, indicating tipping toward tumor tolerance. This is in line with previous reports showing that cLNs influence the development of delayed type hypersensitivity to injected peptides, contributing to tolerance for antigens delivered by nasal route (Wolvers et al., 1999). It has been therefore suggested that cLNs may be more potent inducers of tumor tolerance than other LNs (Harling-Berg et al., 1999; Wolvers et al., 1999; Thomas et al., 2008). In the context of immunotherapy, it is possible that the presence of extracranial tumor overcomes the cLN-induced tumor tolerance by stimulating tumor-specific T cell priming in LNs at locations that better support the development of anti-tumor immune responses (Figure 1).

Several other factors may play an important role in restricting immune responses against sole tumors in the brain. Lymphatic vessels were recently rediscovered at the dura (Aspelund et al., 2015; Louveau et al., 2015b); in contrast, the brain parenchyma lacks classical lymphatic vessels. Soluble antigens from the cerebrospinal fluid (CSF) and brain parenchyma are thought to efficiently drain to the cLNs and different drainage routes have been proposed (Carare et al., 2008; Louveau et al., 2015a, 2017; Engelhardt et al., 2017). While there is substantial evidence that APCs from the CSF also efficiently migrate to the cLNs, migration of APCs from within brain parenchyma to the regional LNs is still a matter of debate (Carare et al., 2008; Louveau et al., 2015a, 2017; Engelhardt et al., 2017). Thus, the (in)ability of APCs to efficiently reach cLNs may impact immune responses in the brain. The abundance of dendritic cells in brain tumors has been also reported to be lower than in s.c. tumors of the same type (Okada et al., 2004). Moreover, characteristics of APCs may differ between intracranial and extracranial tumors.

In the context of immune checkpoint blockade, the access of therapeutic antibodies to tumors in the brain is another consideration. Although the blood-tumor barrier in the brain can be leaky to a variable degree (Lockman et al., 2010; Matthias et al., 2016), it is unclear to what extent immune checkpoint inhibitors enter tumors in the brain directly and how this compares to the extracranial tumor sites. It is possible that therapeutic antibodies are carried into the brain on T cells, as recently shown for anti-PD-1 antibody in extracranial tumors (Arlauckas et al., 2017; Figure 1B).

Another consideration is that metastatic tumors in the brain are genetically and phenotypically different from the primary tumors they originate from, including differences in the expression of immunomodulatory genes (Brastianos et al., 2015; Rippaus et al., 2016), which is likely to influence the immune responses.

Immune checkpoints are key to controlling effector T cell function and consequently anti-tumor immunity. Thus, differences in immune checkpoint expression between BrM and extracranial sites could contribute to differences in therapeutic responses. Clinically, most relevant immune checkpoints are PD-1 and its ligand PD-L1, as well as CTLA-4. A handful of studies investigated expression of these molecules in BrM. PD-1 positive immune cells were found in 3.1–68% of BrM samples, thus showing a large discrepancy in results, with one study reporting the highest PD-1 expression in melanoma BrM (Berghoff et al., 2015, 2016b; Harter et al., 2015). PD-L1 positive immune cells were found in 25–28% of BrM specimens (Berghoff et al., 2016b; Mansfield et al., 2016; Teglasi et al., 2017). There was a wide discrepancy between studies investigating PD-L1 expression on tumor cells, reporting a presence of PD-L1 positive tumor cells in 21.9–75% of BrM specimens (Berghoff et al., 2015, 2016a,b; Harter et al., 2015; Kluger et al., 2015; Mansfield et al., 2016; Ogiya et al., 2017; Takamori et al., 2017, 2018; Teglasi et al., 2017; Zhou et al., 2018). With exception of one study (Berghoff et al., 2016b), the majority of studies showed good agreement between the amount of tumor cells expressing PD-L1 in matched primary tumors and BrM (Ogiya et al., 2017; Takamori et al., 2017; Teglasi et al., 2017). Similar comparison could not been found for PD-1 or CTLA-4. Thus, while several mechanisms have been identified that potentially contribute to the attenuated ability of BrM to induce anti-tumor responses (Figure 1), the evidence for differential expression of immune checkpoints in BrM and primary tumors is currently missing.

## How Does the Immunity Spread From Extracranial to Intracranial Cancer Lesions?

A limited number of studies that investigated the role of extracranial tumor in immunotherapies in BrM determined that T cells are critical for the spread of therapeutic efficacy to the brain. Taggart et al. (2018) demonstrated that CD8+, but not CD4+ T cells are required for inhibition of intracranial B16 melanoma tumors following immune checkpoint blockade in mice bearing simultaneous tumors under the skin (Taggart et al., 2018). In addition, NK cells were critical for intracranial efficacy. In contrast, intracranial rejection of K-1735M2 melanoma tumors following IFNβ-mediated rejection of subcutaneous tumors required both CD4+ and CD8+ T cells (Lu et al., 2003). Thus, while there may be variations between therapeutic modalities and models, T cells seem to be consistently and unsurprisingly required for the spread of immunity to brain tumors. In the presence of subcutaneous B16 melanoma tumors, immune checkpoint blockade leads to a systemic expansion of CD8+ effector T cells and their enhanced trafficking to intracranial tumors (Taggart et al., 2018), thereby spreading anti-tumor immunity into the brain (Figure 1).

## Trafficking of T Cells to Brain Metastases

Efficient trafficking of T cells to tumors is critical for the efficacy of immunotherapies. Trafficking of T cells to primary brain tumors has been extensively reviewed elsewhere (Ratnam et al., 2019). We will here focus on metastatic brain tumors and specifics of T cell homing in a metastatic setting.

Only a few studies investigated trafficking of endogenous T cells to metastatic brain tumors. Calzascia et al. (2005) used M57 fibrosarcoma model which induces spontaneous antitumor immune response. They demonstrated that growth of M57 tumors at different locations induces site-specific expression of adhesion molecules on antigen-specific T cells within respective tumor-draining LNs. Intracranial tumors induced T cell proliferation only in cervical and lumbar LNs, and proliferating T cells upregulated α4β1 integrin, also known as Very late antigen 4 (VLA4), P and E-selectins, and downregulated αEβ7. This expression pattern of adhesion molecules differed from the one induced on T cells by s.c. and intraperitoneal (i.p.) tumor growth within inguinal (iLNs) and mesenteric LNs (mLNs). T cells primed within cLNs homed 2.5-times more efficiently to brain tumors as compared to the iLN-primed T cells, and their homing was α4-dependent, suggesting that site-specific homing phenotype is imprinted on T cells in a tumor location-dependent manner (Calzascia et al., 2005). In further work, the same group demonstrated that during the subsequent effector phase, αEβ7 is upregulated specifically on T cells within brain tumors, but not in s.c. tumors, and promotes T cell retention within the brain (Masson et al., 2007).

In the context of adoptive T cell therapy, T cell polarization has been shown to influence the efficiency of T cell trafficking to brain tumors, due to polarization-specific expression of cell surface adhesion molecules. *In vitro* polarized, ovalbumin-specific (OT-I) Type I cytotoxic T lymphocytes (Tc1) were shown to express higher levels of VLA-4 than Tc2 lymphocytes. This resulted in a significantly enhanced trafficking of adoptively transferred Tc1 versus Tc2 cells to intracranial ovalbumin-expressing M05 melanoma tumors in VLA-4-dependent manner (Sasaki et al., 2007). Homing of Tc1 cells to intracranial tumors was further enhanced by intra-tumoral injection of IFNα-overexpressing dendritic cells in a CXCL10-dependent manner (Nishimura et al., 2006). Similar polarization-dependent trafficking has been reported for CD4+ T cells. Hoepner et al. (2013) demonstrated superior homing of adoptively transferred antigen-specific CD4+ Th1 as compared to Th2 cells to intracranial MC57-GP fibrosarcoma and EG-7 lymphoma tumors.

In line with the observations that extracranial tumor potentiates intracranial efficacy of immunotherapies, the impact of extracranial tumor on T cell homing to brain tumors in the context of immunotherapies and spontaneous antitumor immune responses has also been demonstrated. In our recent study, the presence of s.c. B16 melanoma tumor in addition to the intracranial tumor was required for efficient trafficking of CD8+ T cells to brain tumors following combined PD-1 plus CTLA-4 blockade (Taggart et al., 2018). This is in line with a study using weakly immunogenic lymphoma model, reporting strong increase in CD8+ T cell infiltration into intracranial tumors following concurrent subcutaneous injection of cancer cells, leading to prolonged survival (Thomas et al., 2008). In this study the effect of s.c. cancer cell injection was dose-dependent. Similarly, in a model with concurrent subcutaneous and intracranial EMT6 breast carcinoma tumors, rejection of s.c. tumors through peri-tumoral administration of CpG ODN potentiated the infiltration of CD4+, CD8+ T cells and NK cells into established intracranial tumors (Xiong et al., 2008). In the presence of s.c. B16 melanoma tumors, combined PD-1 plus CTLA-4 blockade resulted in drastic upregulation of T cell entry receptors vascular cell adhesion molecule 1 (VCAM-1) and intercellular adhesion molecule 1 (ICAM-1) on blood vessels within intracranial tumors, which might have contributed to the enhanced T cell infiltration (Taggart et al., 2018).

## T Cells in Human Brain Metastases

Metastatic brain tumors are infiltrated by T cells to a variable degree not only in preclinical models, but also in patients. A number of studies investigated the presence of tumor infiltrating lymphocytes (TIL) in BrM. Immunohistochemical analysis of BrM in a mixed entity cohort of 252 patients revealed three different patterns of infiltration by CD3+ and CD8+ T cells (stromal, peritumoral, diffuse), with highest levels observed in renal cell carcinoma (Harter et al., 2015). In a different cohort, CD3+ T cells were present in 115/116 BrM specimens, while CD8+ T cells were present in 112/116 specimens. The highest density of both cell types was found in melanoma BrM, followed by renal cell cancer and lung cancer BrM (Berghoff et al., 2016a). A study focusing on breast cancer (84 cases) reported an infiltration of BrM by CD4+ and CD8+ T cells in 96 and 98% of cases, respectively. TILs were more abundant in the stroma than in the tumor compartment (Duchnowska et al., 2016). In a cohort of 32 small cell lung cancer (SCLC) BrM specimens, a dense accumulation of CD3+, CD8+, and CD45RO+ T cells in the perivascular area was observed, while FOXP3+ TILs were more abundant within the tumor area and less within the perivascular area (Berghoff et al., 2016b).

Comparative analyses of TIL infiltration at different sites consistently demonstrated a lower presence of TILs in BrM as compared to the primary tumors or extracerebral metastases. A study in metastatic melanoma reported lower abundance of TILs in brain and skin metastases as compared to metastases in the LNs, soft tissue and other extracranial visceral sites (Kluger et al., 2015). A significantly lower abundance of TILs was also reported for BrM originating from lung cancer as compared to the primary lung cancer (Mansfield et al., 2016; Zhou et al., 2018). In breast cancer patients, fewer intratumoral and stromal CD4+ and CD8+ TILs were observed in BrM as compared to the primary tumor (Sobottka et al., 2016; Ogiya et al., 2017) or to metastatic cancer lesions at other sites (Cimino-Mathews et al., 2013).

There is an increasing evidence for the correlation between TIL infiltration in BrM and patient outcomes. In a mixed entity cohort of cancer patients, high amounts of TILs negatively correlated with BrM size (Harter et al., 2015) and the density of CD3+, CD8+, and CD45RO+ TILs showed a positive correlation with favorable median OS times (Berghoff et al., 2016a). In SCLC BrM, the presence of CD45RO+ TILs alone correlated with a significantly longer median survival time compared to patients without the presence of CD45RO+ TILs (Berghoff et al., 2016b). Low versus high stromal CD8+ TIL numbers in BrM were also associated with a significantly shorter OS in lung cancer (Zhou et al., 2018). In breast cancer patients with BrM, the OS was shorter in patients with low TILs as compared to those with high TILs. Moreover, OS following the initial BrM diagnosis was significantly shorter in patients with low TIL counts in BrM specifically in the triple negative breast cancer subgroup (Ogiya et al., 2017).

Unlike TIL infiltration, the association of immune checkpoint expression with patient outcomes is less clear. Three of the studies reported that increased PD-L1 levels on immune (Mansfield et al., 2016) and tumor cells (Kluger et al., 2015), and increased PD-1 levels on tumor cells (Duchnowska et al., 2016) were associated with increased OS. This is in contrast to studies that found no correlation between OS and PD-L1 or PD-1 expression (Berghoff et al., 2015, 2016a; Harter et al., 2015; Teglasi et al., 2017). In a small study PD-L1 expression on tumor cells was even associated with a worse brain-specific disease free survival (Takamori et al., 2018).

In summary, TIL infiltration is frequently found in BrM in patients, however, at a lower rate than in primary tumors and extracerebral metastases. While TIL infiltration in BrM positively correlates with patient outcomes, the role of immune checkpoint expression in this context is less clear.

## Sexual Dimorphism in Immune Checkpoint Inhibitor Therapy

Sexual dimorphism of the immune system is well described, and it is caused by hormonal, genetic, and environmental factors (Mirandola et al., 2015; Klein and Flanagan, 2016; Capone et al., 2018). There is evidence that sexual dimorphism also influences the efficacy of immune checkpoint inhibitor therapy. A recent retrospective study found greater benefit of anti-CTLA-4 therapy in men as compared to women, while no sex-specific differences were observed with anti-PD-1 treatment (Botticelli et al., 2017). Another study reported that low numbers of partially exhausted cytotoxic T lymphocytes correlated with female sex, and in this group of patients a combined PD-1/CTLA-4 blockade resulted in higher overall response rates as compared to anti-PD-1 monotherapy, while no difference between the two treatment regimens was observed in patients with high numbers of partially exhausted cytotoxic T lymphocytes (Loo et al., 2017). Moreover, a preclinical study in B16 melanoma-bearing mice found that tumor growth following PD-L1 blockade was more strongly reduced in female as compared to male animals in a PD-1-independent manner, and this was linked to a stronger reduction in Treg function (Lin et al., 2010). Notably, BrM occur more commonly in males than females, regardless of primary cancer type, age, or region of the world (Sun et al., 2012). Moreover, women with BrM have a longer survival than men. It is thought that sex disparity in the immune responses and in astrocytic production of cytokines may be important underlying factors for the observed differences in the frequency of BrM between sexes.

## Conclusion

Preclinical and clinical studies already revealed numerous critical differences in the immunology of BrM as compared to extracranial cancer lesions, and many more differences are expected to be uncovered. This may require that strategies for the improvement of efficacy of immune checkpoint blockade are tailored according to these differences in order to achieve optimal efficacy in the brain, in addition to extracranial sites. As it has been suggested that priming of immune responses in cLNs induces more potent tumor tolerance than LNs at other sites (Harling-Berg et al., 1999; Wolvers et al., 1999; Thomas et al., 2008), it will be important to investigate differences in processes within LNs at individual anatomical locations during immune checkpoint blockade. Data from healthy brain and non-cancerous CNS disorders suggest that APC migration and antigen drainage to the LNs is less efficient in the brain as compared to extracranial sites (Carare et al., 2008; Louveau et al., 2015a; Engelhardt et al., 2017). Although it is unclear whether this also applies to APCs in the context of brain malignancies, APCs may be another important area for future investigations in the context of immune checkpoint blockade in BrM, with a potential to pinpoint strategies to enhance APC function and migration. Distinct adhesion/homing receptor patterns found on antigen-specific T cells at different anatomical locations (Calzascia et al., 2005; Masson et al., 2007) imply there is an opportunity to improve the efficacy of immune checkpoint inhibitor therapy by enhancing organ-specific T cell trafficking through engineering of T cells to express optimized homing receptor patterns. While it has been shown that immune checkpoint blockade enhances trafficking of T cells to brain tumors (Taggart et al., 2018), it is unclear which molecules and pathways are involved in this process and whether they differ from extracranial sites. The mechanistic role of other immune cells whose activation (NK cells) or infiltration (microglia, macrophages) into intracranial tumors has been shown to be increased following immune checkpoint blockade (Taggart et al., 2018) also remains to be elucidated. Another emerging area in cancer immunotherapy, which has not yet been considered in the context of BrM, is the role of sexual dimorphism (Capone et al., 2018). Addressing these open questions in the specific context of BrM will hopefully enable us to advance immunotherapies for metastatic tumors located in the brain.

## Author Contributions

ML researched the literature on immune crosstalk between brain tumors and the periphery, on factors restricting immune responses against tumors in the brain, on spread of immunity from extracranial to intracranial cancer lesions, on T cell trafficking and sexual dimorphism, wrote the manuscript, and generated the figure. TA researched the literature on T cells in human brain metastases and drafted the corresponding chapter. CF researched the literature on T cell trafficking and sexual dimorphism, and contributed to the writing of the corresponding chapters. FJ researched the literature on immune checkpoints in brain metastases and drafted the corresponding paragraphs.

## Conflict of Interest

The authors declare that the research was conducted in the absence of any commercial or financial relationships that could be construed as a potential conflict of interest.

## References

[B1] Ajithkumar T. Parkinson C. Fife K. Corrie P. Jefferies S. (2015). Evolving treatment options for melanoma brain metastases. *Lancet Oncol.* 16 e486–e497. 10.1016/S1470-2045(15)00141-2 26433822

[B2] Arlauckas S. P. Garris C. S. Kohler R. H. Kitaoka M. Cuccarese M. F. Yang K. S. (2017). In vivo imaging reveals a tumor-associated macrophage-mediated resistance pathway in anti-PD-1 therapy. *Sci. Transl. Med.* 9:eaal3604. 10.1126/scitranslmed.aal3604 28490665 PMC5734617

[B3] Aspelund A. Antila S. Proulx S. T. Karlsen T. V. Karaman S. Detmar M. (2015). A dural lymphatic vascular system that drains brain interstitial fluid and macromolecules. *J. Exp. Med.* 212 991–999. 10.1084/jem.20142290 26077718 PMC4493418

[B4] Barth R. F. Kaur B. (2009). Rat brain tumor models in experimental neuro-oncology: the C6, 9L, T9, RG2, F98, BT4C, RT-2 and CNS-1 gliomas. *J. Neurooncol.* 94 299–312. 10.1007/s11060-009-9875-7 19381449 PMC2730996

[B5] Berghoff A. S. Fuchs E. Ricken G. Mlecnik B. Bindea G. Spanberger T. (2016a). Density of tumor-infiltrating lymphocytes correlates with extent of brain edema and overall survival time in patients with brain metastases. *Oncoimmunology* 5:e1057388. 10.1080/2162402x.2015.1057388 26942067 PMC4760339

[B6] Berghoff A. S. Ricken G. Wilhelm D. Rajky O. Widhalm G. Dieckmann K. (2016b). Tumor infiltrating lymphocytes and PD-L1 expression in brain metastases of small cell lung cancer (SCLC). *J. Neurooncol.* 130 19–29. 10.1007/s11060-016-2216-8 27436101

[B7] Berghoff A. S. Ricken G. Widhalm G. Rajky O. Dieckmann K. Birner P. (2015). Tumour-infiltrating lymphocytes and expression of programmed death ligand 1 (PD-L1) in melanoma brain metastases. *Histopathology* 66 289–299. 10.1111/his.12537 25314639

[B8] Bidoli R. C. P. Catino A. Grossi F. Noberasco C. Gelsomino F. Gilli M. (2016). Efficacy and safety data from patients with advanced squamous NSCLC and brain metastases participating in the nivolumab expanded access programme (EAP) in Italy. *Ann. Oncol.* 27 v460–v496.

[B9] Botticelli A. Onesti C. E. Zizzari I. Cerbelli B. Sciattella P. Occhipinti M. (2017). The sexist behaviour of immune checkpoint inhibitors in cancer therapy? *Oncotarget* 8 99336–99346. 10.18632/oncotarget.22242 29245905 PMC5725096

[B10] Brastianos P. K. Carter S. L. Santagata S. Cahill D. P. Taylor-Weiner A. Jones R. T. (2015). Genomic characterization of brain metastases reveals branched evolution and potential therapeutic targets. *Cancer Discov.* 5 1164–1177. 10.1158/2159-8290.CD-15-0369 26410082 PMC4916970

[B11] Calzascia T. Masson F. Di Berardino-Besson W. Contassot E. Wilmotte R. Aurrand-Lions M. (2005). Homing phenotypes of tumor-specific CD8 T cells are predetermined at the tumor site by crosspresenting APCs. *Immunity* 22 175–184. 10.1016/j.immuni.2004.12.008 15723806

[B12] Capone I. Marchetti P. Ascierto P. A. Malorni W. Gabriele L. (2018). Sexual dimorphism of immune responses: a new perspective in cancer immunotherapy. *Front. Immunol.* 9:552. 10.3389/fimmu.2018.00552 29619026 PMC5871673

[B13] Carare R. O. Bernardes-Silva M. Newman T. A. Page A. M. Nicoll J. A. Perry V. H. (2008). Solutes, but not cells, drain from the brain parenchyma along basement membranes of capillaries and arteries: significance for cerebral amyloid angiopathy and neuroimmunology. *Neuropathol. Appl. Neurobiol.* 34 131–144. 10.1111/j.1365-2990.2007.00926.x 18208483

[B14] Cimino-Mathews A. Ye X. Meeker A. Argani P. Emens L. A. (2013). Metastatic triple-negative breast cancers at first relapse have fewer tumor-infiltrating lymphocytes than their matched primary breast tumors: a pilot study. *Hum. Pathol.* 44 2055–2063. 10.1016/j.humpath.2013.03.010 23701942 PMC3758372

[B15] De Giorgi U. Carteni G. Giannarelli D. Basso U. Galli L. Cortesi E. (2019). Safety and efficacy of nivolumab for metastatic renal cell carcinoma: real-world results from an expanded access programme. *BJU Int.* 123 98–105. 10.1111/bju.14461 29956884

[B16] DeAngelis L. M. (2008). Treatment of brain metastasis. *J. Support. Oncol.* 2008 87–88. 15524068

[B17] Doron H. Pukrop T. Erez N. (2019). A blazing landscape: neuroinflammation shapes brain metastasis. *Cancer Res.* 79 423–436. 10.1158/0008-5472.CAN-18-1805 30679177 PMC6420077

[B18] Duchnowska R. Peksa R. Radecka B. Mandat T. Trojanowski T. Jarosz B. (2016). Immune response in breast cancer brain metastases and their microenvironment: the role of the PD-1/PD-L axis. *Breast Cancer Res.* 18:43. 10.1186/s13058-016-0702-8 27117582 PMC4847231

[B19] Engelhardt B. Vajkoczy P. Weller R. O. (2017). The movers and shapers in immune privilege of the CNS. *Nat. Immunol.* 18 123–131. 10.1038/ni.3666 28092374

[B20] Gerrard G. E. Franks K. N. (2004). Overview of the diagnosis and management of brain, spine, and meningeal metastases. *J. Neurol. Neurosurg. Psychiatry* 75 (Suppl. 2), 37–42. 10.1136/jnnp.2004.040493 PMC1765655 15146038

[B21] Goldberg S. B. Gettinger S. N. Mahajan A. Chiang A. C. Herbst R. S. Sznol M. (2016). Pembrolizumab for patients with melanoma or non-small-cell lung cancer and untreated brain metastases: early analysis of a non-randomised, open-label, phase 2 trial. *Lancet Oncol.* 17 976–983. 10.1016/S1470-2045(16)30053-5 27267608 PMC5526047

[B22] Gonzalez-Cao M. Arance A. Piulats J. M. Marquez-Rodas I. Manzano J. L. Berrocal A. (2017). Pembrolizumab for advanced melanoma: experience from the Spanish Expanded Access Program. *Clin. Transl. Oncol.* 19 761–768. 10.1007/s12094-016-1602-1 28054320

[B23] Harling-Berg C. J. Park T. J. Knopf P. M. (1999). Role of the cervical lymphatics in the Th2-type hierarchy of CNS immune regulation. *J. Neuroimmunol.* 101 111–127. 10.1016/s0165-5728(99)00130-7 10580795

[B24] Harter P. N. Bernatz S. Scholz A. Zeiner P. S. Zinke J. Kiyose M. (2015). Distribution and prognostic relevance of tumor-infiltrating lymphocytes (TILs) and PD-1/PD-L1 immune checkpoints in human brain metastases. *Oncotarget* 6 40836–40849. 10.18632/oncotarget.5696 26517811 PMC4747372

[B25] Hoepner S. Loh J. M. Riccadonna C. Derouazi M. Maroun C. Y. Dietrich P. Y. (2013). Synergy between CD8 T cells and Th1 or Th2 polarised CD4 T cells for adoptive immunotherapy of brain tumours. *PLoS One* 8:e63933. 10.1371/journal.pone.0063933 23717511 PMC3662716

[B26] Kamphorst A. O. Ahmed R. (2013). Manipulating the PD-1 pathway to improve immunity. *Curr. Opin. Immunol* 25 381–388. 10.1016/j.coi.2013.03.003 23582509 PMC5946314

[B27] Kida Y. Cravioto H. Hochwald G. M. Hochgeschwender U. Ransohoff J. (1983). Immunity to transplantable nitrosourea-induced neurogenic tumors. II. Immunoprophylaxis of tumors of the brain. *J. Neuropathol. Exp. Neurol.* 42 122–135. 10.1097/00005072-198303000-00002 6600780

[B28] Klein S. L. Flanagan K. L. (2016). Sex differences in immune responses. *Nat. Rev. Immunol.* 16 626–638. 10.1038/nri.2016.90 27546235

[B29] Kluger H. M. Zito C. R. Barr M. L. Baine M. K. Chiang V. L. Sznol M. (2015). Characterization of PD-L1 expression and associated T-cell infiltrates in metastatic melanoma samples from variable anatomic sites. *Clin. Cancer Res.* 21 3052–3060. 10.1158/1078-0432.CCR-14-3073 25788491 PMC4490112

[B30] Lin P. Y. Sun L. Thibodeaux S. R. Ludwig S. M. Vadlamudi R. K. Hurez V. J. (2010). B7-H1-dependent sex-related differences in tumor immunity and immunotherapy responses. *J. Immunol.* 185 2747–2753. 10.4049/jimmunol.1000496 20686128 PMC3244840

[B31] Lockman P. R. Mittapalli R. K. Taskar K. S. Rudraraju V. Gril B. Bohn K. A. (2010). Heterogeneous blood-tumor barrier permeability determines drug efficacy in experimental brain metastases of breast cancer. *Clin. Cancer Res.* 16 5664–5678. 10.1158/1078-0432.CCR-10-1564 20829328 PMC2999649

[B32] Long G. V. Atkinson V. Menzies A. M. Lo S. Guminski A. D. Brown M. P. (2017). A randomized phase II study of nivolumab or nivolumab combined with ipilimumab in patients (pts) with melanoma brain metastases (mets): the anti-pd1 brain collaboration (ABC). *J. Clin. Oncol.* 35 (Suppl. 15):9508. 10.1200/jco.2017.35.15_suppl.9508

[B33] Loo K. Tsai K. K. Mahuron K. Liu J. Pauli M. L. Sandoval P. M. (2017). Partially exhausted tumor-infiltrating lymphocytes predict response to combination immunotherapy. *JCI Insight* 2L93433. 10.1172/jci.insight.93433 28724802 PMC5518562

[B34] Louveau A. Harris T. H. Kipnis J. (2015a). Revisiting the mechanisms of CNS immune privilege. *Trends Immunol.* 36 569–577. 10.1016/j.it.2015.08.006 26431936 PMC4593064

[B35] Louveau A. Smirnov I. Keyes T. J. Eccles J. D. Rouhani S. J. Peske J. D. (2015b). Structural and functional features of central nervous system lymphatic vessels. *Nature* 523 337–341. 10.1038/nature14432 26030524 PMC4506234

[B36] Louveau A. Plog B. A. Antila S. Alitalo K. Nedergaard M. Kipnis J. (2017). Understanding the functions and relationships of the glymphatic system and meningeal lymphatics. *J. Clin. Invest.* 127 3210–3219. 10.1172/JCI90603 28862640 PMC5669566

[B37] Lu W. Su J. Kim L. S. Bucana C. D. Donawho C. He J. (2003). Active specific immunotherapy against occult brain metastasis. *Cancer Res.* 63 1345–1350. 12649197

[B38] Mansfield A. S. Aubry M. C. Moser J. C. Harrington S. M. Dronca R. S. Park S. S. (2016). Temporal and spatial discordance of programmed cell death-ligand 1 expression and lymphocyte tumor infiltration between paired primary lesions and brain metastases in lung cancer. *Ann. Oncol.* 27 1953–1958. 10.1093/annonc/mdw289 27502709 PMC5035793

[B39] Margolin K. Ernstoff M. S. Hamid O. Lawrence D. McDermott D. Puzanov I. (2012). Ipilimumab in patients with melanoma and brain metastases: an open-label, phase 2 trial. *Lancet Oncol.* 13 459–465. 10.1016/S1470-2045(12)70090-6 22456429

[B40] Masson F. Calzascia T. Di Berardino-Besson W. de Tribolet N. Dietrich P. Y. Walker P. R. (2007). Brain microenvironment promotes the final functional maturation of tumor-specific effector CD8+ T cells. *J. Immunol.* 179 845–853. 10.4049/jimmunol.179.2.845 17617575

[B41] Matthias O. Jonas B. Yunxiang L. Gergely S. Miriam G. Berghoff A. S. (2016). Impact of blood–brain barrier integrity on tumor growth and therapy response in brain metastases. *Clin. Cancer Res.* 22 6078–6087. 10.1158/1078-0432.ccr-16-1327 27521448

[B42] Medawar P. B. (1948). Immunity to homologous grafted skin; the fate of skin homografts transplanted to the brain, to subcutaneous tissue, and to the anterior chamber of the eye. *Br. J. Exp. Pathol.* 29 58–69. 18865105 PMC2073079

[B43] Mirandola L. Wade R. Verma R. Pena C. Hosiriluck N. Figueroa J. A. (2015). Sex-driven differences in immunological responses: challenges and opportunities for the immunotherapies of the third millennium. *Int. Rev. Immunol.* 34 134–142. 10.3109/08830185.2015.1018417 25901858

[B44] Murphy J. B. Sturm E. (1923). Conditions determining the transplantability of tissues in the brain. *J. Exp. Med.* 38 183–197. 10.1084/jem.38.2.183 19868782 PMC2128434

[B45] Nishimura F. Dusak J. E. Eguchi J. Zhu X. Gambotto A. Storkus W. J. (2006). Adoptive transfer of type 1 CTL mediates effective anti-central nervous system tumor response: critical roles of IFN-inducible protein-10. *Cancer Res.* 66 4478–4487. 10.1158/0008-5472.can-05-3825 16618775

[B46] Ogiya R. Niikura N. Kumaki N. Yasojima H. Iwasa T. Kanbayashi C. (2017). Comparison of immune microenvironments between primary tumors and brain metastases in patients with breast cancer. *Oncotarget* 8 103671–103681. 10.18632/oncotarget.22110 29262592 PMC5732758

[B47] Okada H. Tsugawa T. Sato H. Kuwashima N. Gambotto A. Okada K. (2004). Delivery of interferon-alpha transfected dendritic cells into central nervous system tumors enhances the antitumor efficacy of peripheral peptide-based vaccines. *Cancer Res.* 64 5830–5838. 10.1158/0008-5472.can-04-0130 15313927

[B48] Parakh S. Park J. J. Mendis S. Rai R. Xu W. Lo S. (2017). Efficacy of anti-PD-1 therapy in patients with melanoma brain metastases. *Br. J. Cancer* 116 1558–1563. 10.1038/bjc.2017.142 28524161 PMC5518864

[B49] Paul D. B. Barth R. F. Yang W. Shen G. H. Kim J. Triozzi P. L. (2000). B7.1 expression by the weakly immunogenic F98 rat glioma does not enhance immunogenicity. *Gene Ther.* 7 993–999. 10.1038/sj.gt.3301209 10871746

[B50] Puzanov I. Wolchok J. D. Ascierto P. A. Hamid O. Kim M. (2013). Anti-CTLA-4 and BRAF Inhibition in patients with metastatic melanoma and brain metastases. *Expert Rev. Dermatol.* 8 479–487. 10.1586/17469872.2013.835922

[B51] Ratnam N. M. Gilbert M. R. Giles A. J. (2019). Immunotherapy in CNS cancers: the role of immune cell trafficking. *Neuro Oncol.* 21 37–46. 10.1093/neuonc/noy084 29771386 PMC6303437

[B52] Rippaus N. Taggart D. Williams J. Andreou T. Wurdak H. Wronski K. (2016). Metastatic site-specific polarization of macrophages in intracranial breast cancer metastases. *Oncotarget* 7 41473–41487. 10.18632/oncotarget.9445 27203741 PMC5173073

[B53] Sasaki K. Zhu X. Vasquez C. Nishimura F. Dusak J. E. Huang J. (2007). Preferential expression of very late antigen-4 on type 1 CTL cells plays a critical role in trafficking into central nervous system tumors. *Cancer Res.* 67 6451–6458. 10.1158/0008-5472.can-06-3280 17616706

[B54] Shirai Y. (1921). On the transplantation of the rat sarcoma in adult heterogeneous animals. *Jap. Med. World* 1 14–15. 10.1038/nm.4041 26855149

[B55] Sobottka B. Pestalozzi B. Fink D. Moch H. Varga Z. (2016). Similar lymphocytic infiltration pattern in primary breast cancer and their corresponding distant metastases. *Oncoimmunology* 5:e1153208. 10.1080/2162402X.2016.1153208 27471624 PMC4938373

[B56] Su M. Y. Taylor J. A. Villarreal L. P. Nalcioglu O. (2000). Prediction of gene therapy-induced tumor size changes by the vascularity changes measured using dynamic contrast-enhanced MRI. *Magn. Reson. Imaging* 18 311–317. 10.1016/s0730-725x(00)00119-3 10745141

[B57] Sun T. Warrington N. M. Rubin J. B. (2012). Why does Jack, and not Jill, break his crown? Sex disparity in brain tumors. *Biol. Sex. Differ.* 3:3. 10.1186/2042-6410-3-3 22277186 PMC3293746

[B58] Taggart D. Andreou T. Scott K. J. Williams J. Rippaus N. Brownlie R. J. (2018). Anti-PD-1/anti-CTLA-4 efficacy in melanoma brain metastases depends on extracranial disease and augmentation of CD8(+) T cell trafficking. *Proc. Natl. Acad. Sci. U.S.A.* 115 E1540–E1549. 10.1073/pnas.1714089115 29386395 PMC5816160

[B59] Takamori S. Toyokawa G. Okamoto I. Takada K. Kinoshita F. Kozuma Y. (2018). Clinical significance of PD-L1 expression in brain metastases from non-small cell lung cancer. *Anticancer Res.* 38 553–557. 29277823 10.21873/anticanres.12258

[B60] Takamori S. Toyokawa G. Okamoto I. Takada K. Kozuma Y. Matsubara T. (2017). Discrepancy in Programmed Cell Death-Ligand 1 Between Primary and Metastatic Non-small Cell Lung Cancer. *Anticancer Res.* 37 4223–4228. 28739710 10.21873/anticanres.11813

[B61] Tawbi P. F. H. Algazi A. Hamid O. Hodi F. S. Moschos S. Thomas R. P. (2017). Efficacy and safety of nivolumab plus ipilimumab in patients with melanoma metastatic to the brain: results of the phase II Study checkmate 204. *J. Clin. Oncol.* 35 (Suppl. 15):9507. 10.1056/NEJMoa1805453 30134131 PMC8011001

[B62] Teglasi V. Reiniger L. Fabian K. Pipek O. Csala I. Bago A. G. (2017). Evaluating the significance of density, localization, and PD-1/PD-L1 immunopositivity of mononuclear cells in the clinical course of lung adenocarcinoma patients with brain metastasis. *Neuro Oncol.* 19 1058–1067. 10.1093/neuonc/now309 28201746 PMC5570158

[B63] Thomas D. L. Kranz D. M. Roy E. J. (2008). Experimental manipulations of afferent immune responses influence efferent immune responses to brain tumors. *Cancer Immunol. Immunother.* 57 1323–1333. 10.1007/s00262-008-0467-8 18278494 PMC11030392

[B64] Valiente M. Ahluwalia M. S. Boire A. Brastianos P. K. Goldberg S. B. Lee E. Q. (2018). The evolving landscape of brain metastasis. *Trends Cancer* 4 176–196. 10.1016/j.trecan.2018.01.003 29506669 PMC6602095

[B65] Volovitz I. Marmor Y. Azulay M. Machlenkin A. Goldberger O. Mor F. (2011). Split immunity: immune inhibition of rat gliomas by subcutaneous exposure to unmodified live tumor cells. *J. Immunol.* 187 5452–5462. 10.4049/jimmunol.1003946 21998458

[B66] Walker L. S. Sansom D. M. (2011). The emerging role of CTLA4 as a cell-extrinsic regulator of T cell responses. *Nat. Rev. Immunol.* 11 852–863. 10.1038/nri3108 22116087

[B67] Wei S. C. Duffy C. R. Allison J. P. (2018). Fundamental mechanisms of immune checkpoint blockade therapy. *Cancer Discov.* 8 1069–1086. 10.1158/2159-8290.CD-18-0367 30115704

[B68] Wolvers D. A. Coenen-de Roo C. J. Mebius R. E. van der Cammen M. J. Tirion F. Miltenburg A. M. (1999). Intranasally induced immunological tolerance is determined by characteristics of the draining lymph nodes: studies with OVA and human cartilage gp-39. *J. Immunol.* 162 1994–1998. 9973470

[B69] Xiong Z. Gharagozlou S. Vengco I. Chen W. Ohlfest J. R. (2008). Effective CpG immunotherapy of breast carcinoma prevents but fails to eradicate established brain metastasis. *Clin. Cancer Res.* 14 5484–5493. 10.1158/1078-0432.CCR-07-4139 18765540

[B70] Zhou J. Gong Z. Jia Q. Wu Y. Yang Z. Z. Zhu B. (2018). Programmed death ligand 1 expression and CD8(+) tumor-infiltrating lymphocyte density differences between paired primary and brain metastatic lesions in non-small cell lung cancer. *Biochem. Biophys. Res. Commun.* 498 751–757. 10.1016/j.bbrc.2018.03.053 29526752

